# COVID-19 Literature Published in Emergency Medicine Journals in 2020

**DOI:** 10.5811/westjem.2022.1.55029

**Published:** 2022-05-05

**Authors:** Ching-Hsing Lee

**Affiliations:** Chang Gung University College of Medicine, Chang Gung Memorial Hospital, Department of Emergency Medicine, Keelung, Taiwan

## Abstract

**Introduction:**

Coronavirus disease 2019 (COVID-19)-related articles published in emergency medicine (EM) journals provide insight into the responses of EM researchers and journal editors globally to a newly emerging infectious disease. We studied trends in the number, types, and national origins of COVID-19 literature published in EM journals to investigate knowledge transmission via scientific publication during the pandemic.

**Methods:**

This was a retrospective observational study. The EM journal list was adopted from the 2019 Journal Citation Reports. We retrieved data from the SCOPUS database, limited to publication year 2020, and identified COVID-19 publications when the title, abstract, or keywords included “COVID” or “SARS.” The outcome measurements were as follows: 1) monthly COVID-19 publication numbers in EM journals; 2) the percentage of COVID-19 published literature in terms of total journal publications; 3) the countries, affiliations, and authors of COVID-19-related publications; 4) the differences in the proportions of “Articles” and “Letters” between COVID-19 and non-COVID-19 publications; and 5) the total, average, and maximum number of times cited for different types of COVID-19-related scientific literature.

**Results:**

We retrieved a total of 7,457 published papers from 31 EM journals. There were 765 (10.26%) COVID-19-related publications in 27 journals contributed by 67 countries; the first authors were from 49 countries. The monthly COVID-19 publication numbers in the categories of “Letters” and “Articles” were nearly equal before July 2020. The yearly proportions of COVID-19-focused articles and letters were 48.8% and 29.9%, respectively, while non-COVID-19 proportions were 72.1% and 9.8%, respectively. The chi-squared statistic of the differences between the numbers of articles and letters in COVID-19 and non-COVID-19 published research was significant (P < .001).

**Conclusion:**

An analysis of COVID-19 publications in EM journals indicated that, in the early stage of a newly emerging infectious disease, the number of letters and articles increased simultaneously. The proportion of COVID-19-focused letters was higher than those published on other topics. The “Article” and “Review” category of COVID-19 research was cited more times than that of “Letters.”

## INTRODUCTION

The World Health Organization (WHO) issued a media statement on cases of “viral pneumonia” reported by the Municipal Health Commission of Wuhan, People’s Republic of China, on December 31, 2019.^1^ The WHO Director-General declared the coronavirus disease 2019 (COVID-19) outbreak a public health emergency of international concern (PHEIC), the highest level of alarm, on January 30, 2020.^1^ One year after the PHEIC declaration, the global number of COVID-19 cases exceeded 100 million on January 28, 2021. 2 The COVID-19 pandemic has imposed an enormous strain on global healthcare systems, especially on frontline workers in emergency departments (ED).^3^,^4^ The pandemic affects clinical practice, medical education, and academic publications.^5^,^6^ COVID-19-related publications in emergency medicine (EM) journals provide insights into the responses of EM journals and researchers to a newly emerging infectious disease. Interactions between the emerging disease, EM researchers, and journal editorial boards revealed the process of knowledge transmission from the perspective of the scientific publication format during the COVID-19 pandemic. This will be of interest to future researchers and journal editors.

In this study we investigated trends of COVID-19-related publications in EM journals in 2020 by evaluating the publication numbers, type, country of authorship, affiliation, authors, and the number of times cited. Our goal was to facilitate knowledge transfer by guiding researchers who aim to submit COVID-19-related research.

## METHODS

### Study Design and Setting

This was a retrospective observational study. As no human subjects were involved, we received a waiver from our institutional review board. The EM journal list was adopted from that of the 2019 Journal Citation Reports (JCR) (Appendix 1).^7^ We retrieved the publication data from the SCOPUS database.^8^ Journals that were not indexed in the SCOPUS database were accessed via PubMed.^9^

### Publication Selection

We defined COVID-19-related publications as those in which the title, abstract, or keywords included “COVID” or “SARS.” The EM journals were those categorized in the 2019 JCR, and the publication year was limited to 2020. The publication types were adopted from the classification of the SCOPUS database. We defined the country of origin by the nationality of the first author. Author affiliations and details were retrieved by searching SCOPUS.

We retrieved all EM journal publications and COVID-19-focused publications from the SCOPUS database on December 25, 2020, using each journal’s International Standard Serial Number (ISSN). The search terms for COVID-19-related publications and total EM journal publications are listed in Appendix 2. Information on the journals, first-author nationalities, number of times cited, and publication types were recorded.

### Outcome Measurements

The primary outcomes of this study were the chronological trends in COVID-19 commentary and research of different types, which indicated the response times of researchers and EM journals to a newly emerging infectious disease from the perspective of academic research. The secondary outcome measurements were as follows: 1) monthly COVID-19 publication numbers in EM journals; 2) percentages of COVID-19-focused publications in terms of total journal publications; 3) the leading countries, affiliations, and authors in terms of COVID-19 publication numbers; 4) differences between the proportions of “Articles” and “Letters” in COVID-19 and non-COVID-19 published literature; and (5) total, average, and the maximum number of times cited for the different types of COVID-19 publications.

**Population Health Research Capsule**

What do we already know about this issue?

*The COVID-19 pandemic has affected many aspects of the medical community, including clinical practice, medical education, and published research.*

What was the research question?

*What were the impacts of COVID-19 on published scientific literature in EM journals in 2020?*

What was the major finding of the study?

*We identified trends in the number and types of publications, authorship, and citations.*

How does this improve population health?

*This study guides researchers on how to facilitate knowledge transfer via scientific publication during a pandemic.*

### Analysis

We analyzed the distributions of publication numbers and the number of times cited using descriptive statistics. The differences between the numbers of “Articles” and “Letters” of COVID-19-focused and non- COVID-19 publications were analyzed with the chi-squared statistic. We performed all analyses using SAS statistical software version 9.2 (SAS Institute, Cary, NC).

### Patient and Public Involvement

No patients were involved in this study.

## RESULTS

We retrieved a total of 7,457 publications in 2020 from 31 EM journals. There were 765 COVID-19-related publications in 27 journals (10.26% of a total of 31 EM journal publications, 10.63% of 27 journals publishing COVID-19 publications). The monthly COVID-19 publication numbers in 27 EM journals are shown in Table 1. The *American Journal of Emergency Medicine* (*AJEM*) published the most COVID-19-related research (183, 23.9% of the total) followed by the *Western Journal of Emergency Medicine* (*WJEM*) (66, 8.6%) and *Resuscitation* (57, 7.5%). The *WJEM* published the highest proportion of COVID-19-related publications (30.4%), followed by *Emergencias* (26.0%) and the *Emergency Medicine Journal* (20.5%). COVID-19-related publications were contributed by 67 countries and the first authors were from 49 countries.

**Figure 1:**
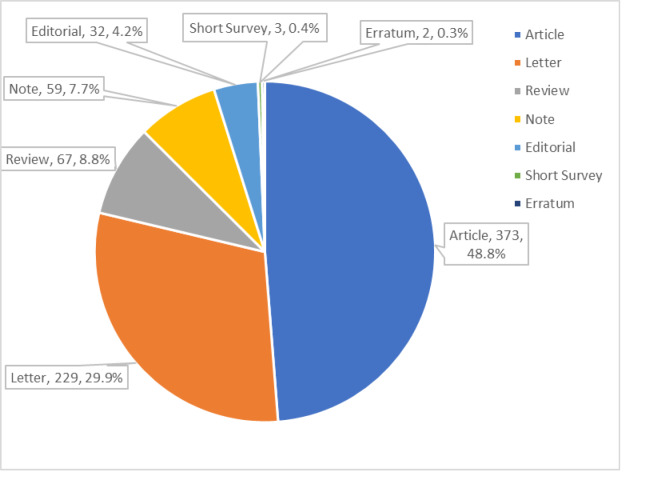
Coronavirus disease 2019 (COVID-19) publication types, publication numbers, and proportions of all COVID-19 publications in emergency medicine journals in 2020.

The leading countries, affiliations, and authors of COVID-19-related publications are listed in Table 2. The publication numbers and percentages of COVID-19 publication types are shown in Figure 1. The three leading types of COVID-19- and non-COVID-19-related publications were identical. Of the 6,692 non-COVID-19 publications, 4825 (72.1%) were articles, 658 (9.8%) were letters, and 510 (7.6%) were reviews. The chi-squared statistic of the differences between the proportions of articles and letters for COVID-19 and non-COVID-19 publications (X^2^ = 295.3) was significant (*P* < .001).

**Figure 2:**
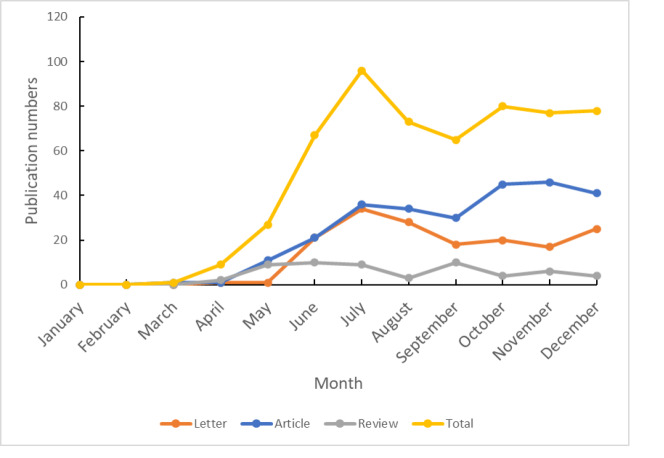
Monthly numbers of three major types of published research and all coronavirus disease 2019-related publications in 2020 in emergency medicine journals.

The monthly numbers of articles, letters, and reviews, and total COVID-19-related publications are shown in Figure 2. The monthly numbers of COVID-19-related letters and articles were nearly equal before July 2020. Then, the number of letters decreased gradually and the number of articles increased steadily after August 2020. The analysis of the number of times COVID-19 publications were cited is shown in Table 3. The most highly cited publications were articles and reviews, which were cited 118 times. The publication type with the highest average number of times cited was review, followed by article. Nearly 60% of review and more than 40% of article and letter publications were cited within one year of publication.

## DISCUSSION

Correct, efficient, and timely knowledge transmission is crucial to healthcare clinicians. In the COVID-19 pandemic era, these components led to high-quality patient care and the enhanced safety of healthcare workers. In addition to peer-reviewed scientific publications, a variety of methods is available to communicate scientific knowledge during a pandemic, including social media, virtual conferencing, and free, open-access medical education.^10^–^12^ Our study focused on the response of EM journals to a newly emerging infectious disease pandemic from the perspective of publications.

The first COVID-19-related publications appeared in March 2020, three months after the emergence of COVID-19, and more than 10% of all EM journal publications in 2020 were related to COVID-19, suggesting that COVID-19 is a rapidly emerging field in EM scientific publication. Twenty-seven of 31 journals (87.1%) published COVID-19-related publications, indicating that the preferences and scope of EM journals are diverse; not all journals publish COVID-19-related research. The majority of the editorial boards of EM journals responded rapidly and were willing to accept newly emerging, important topics. The errata and withdrawal of COVID-19-related publications in these journals will be continuously evaluated in the coming years to clarify the effect of the rapid publishing process on reporting inaccurate, inappropriate, or potentially harmful information.

In the analysis of authorship, the first authors of COVID-19-related publications in EM journals were from 49 countries. The leading five countries (5/49, 10.2%) accounted for 62% of all COVID-19-related publications. The United States was the major contributor of COVID-19 research to EM journals. The five leading affiliations were in the United States, Canada, and Australia. These results suggest that these three countries, their affiliations, and researchers responded rapidly on research design, execution, and publication.

Our analysis of publication type showed that almost 30% of COVID-19-related publications were in the category “Letters” and that the proportion of letters in non-COVID-19-related publications was 9.8%. The difference may reflect the fact that our early understanding of a newly important emerging disease was limited, and there was not enough time, research resources, or clinical cases to conduct an original study. When there are enough resources, cases, and time to allow studies, the proportion of articles would be expected to increase, and that of letters would tend to decrease to baseline. This phenomenon may be due to the response of medical journals to a rapidly emerging new field with a high disease burden. Whether this is true of other specialty journals deserves further study.

The number of times cited analysis provided another perspective on the impact of COVID-19 publications, in addition to the number of publications. The publications-cited results suggested that the research was related to, or the foundation for, another study. Publications that influenced other studies were cited more frequently. The most frequently cited COVID-19 publications and those cited an average number of times were in the categories “Articles” and “Reviews.” The letter type of publication was second in publication number, but the average number of times cited was less than for articles and reviews. The article- and review-formatted publications were more influential than published letters from the perspective of times cited as the measurement of impact. Nearly half of the COVID-19-related publications in 2020 were cited at least once by the end of 2020.

The JCR database from which we adopted the EM journal list is one of the most popular academic publication indexing databases. The SCImago Journal & Country Ranking based on the Scopus database also provides a list of EM journals that is more comprehensive, providing extended coverage on non-English language journals; it indexes 85 EM journals.^13^ Further studies of EM journals not included in this study could provide a more comprehensive viewpoint on the publication trend. The journal publishing policy on free, open-access publications and pre-printed databases for COVID-19-related research may also influence researchers’ options on submitting to journals. These policies facilitate transmission of knowledge during the pandemic era. The article processing charge (APC) is another factor that negatively affects researchers from developing and low-and-middle-income countries who may be unable to pay the publication fees. Future studies on more non-English language journals and the influence of free, open-access policy and APC will help us to understand changes in the publication process of COVID-19-related research in academic EM.

## LIMITATIONS

There are some limitations to this study. First, EM-related research may appear in non-EM specialty journals as well. The overall response of global EM researchers to COVID-19 could be more comprehensively understood if COVID-19-related publications affiliated with EDs that were published in other scientific journals were included. Second, the response of EM journals to COVID-19 is ongoing; the pandemic is not over. Continuous analysis of publication trends in 2021 would provide a more comprehensive view. Third, this pandemic originated from a non-English speaking country, but no EM journals from the 2019 JCR were in Chinese. This result may underestimate the contribution from EM researchers who published in Chinese EM journals during the early stage of the pandemic.

## CONCLUSION

COVID-19-related publications appeared in EM journals within three months of pandemic commencement. The scope of EM journals is wide-ranging and diverse; not all EM journals publish COVID-19 research. In the early stage of a newly emerging infectious disease, the number of letter- and article-format publications increased in parallel, in EM journals. Articles and reviews were cited more frequently than letters. A future study that examines a broader database and the effect of open-access journals and article processing charges on publications in newly emerging fields from developing and low-and-middle-income countries may enhance our understanding of publication trends in EM journals.

## Supplementary Information





## Figures and Tables

**Table 1:** Monthly COVID-19 publication numbers in 2020 in emergency medicine journals (listed in alphabetical order).

Journals	Jan.	Feb.	Mar.	Apr.	May	Jun.	Jul.	Aug.	Sep.	Oct.	Nov.	Dec.	In Press	Total	Journal total publications	% of total publication
*Academic Emergency Medicine*	0	0	0	4	2	5	4	9	5	3	7	4	1	44	309	14.2%
*American Journal of Emergency Medicine*	0	0	0	0	0	1	25	1	6	20	10	9	111	183	1165	15.7%
*Annals of Emergency Medicine*	0	0	0	0	0	5	3	0	4	13	3	5	4	37	421	8.8%
*BMC Emergency Medicine*	0	0	0	0	0	0	0	0	0	1	0	2	0	3	95	3.2%
*Canadian Journal of Emergency Medicine*	0	0	0	0	0	0	13	0	15	0	7	0	4	39	193	20.2%
*EMA - Emergency Medicine Australasia*	0	0	0	0	0	2	0	8	0	11	0	9	9	39	280	13.9%
*Emergencias*	0	0	0	0	0	5	0	14	0	5	0	10	0	34	131	26.0%
*Emergency Medicine Journal*	0	0	0	0	0	4	11	5	4	5	5	3	12	49	239	20.5%
*European Journal of Emergency Medicine*	0	0	0	0	0	7	0	0	0	0	0	0	8	15	138	10.9%
*European Journal of Trauma and Emergency Surgery*	0	0	0	0	0	3	0	4	0	1	0	0	5	13	405	3.2%
*Hong Kong Journal of Emergency Medicine*	0	0	0	0	0	0	0	0	2	0	1	0	2	5	129	3.9%
*Injury*	0	0	0	1	1	2	5	3	0	7	1	6	3	29	840	3.5%
*Journal of Emergency Medicine*	0	0	0	0	1	0	2	0	2	2	7	5	7	26	512	5.1%
*Journal of Emergency Nursing*	0	0	0	0	0	0	0	0	2	0	5	0	1	8	164	4.9%
*Notarzt*	0	0	0	0	0	1	0	1	0	2	0	0	0	4	64	6.3%
*Notfall und Rettungsmedizin*	0	0	0	0	2	8	0	4	2	0	1	3	5	25	155	16.1%
*Pediatric Emergency Care*	0	0	0	0	0	2	0	1	0	4	4	0	0	11	251	4.4%
*Prehospital and Disaster Medicine*	0	0	0	1	0	1	0	13	0	0	0	6	6	27	149	18.1%
*Prehospital Emergency Care*	0	0	0	0	0	0	0	0	0	0	0	0	8	8	189	4.2%
*Resuscitation*	0	0	0	0	0	10	10	8	5	4	10	6	4	57	515	11.1%
*Scandinavian Journal of Trauma, Resuscitation and Emergency Medicine*	0	0	0	0	3	1	1	2	1	2	1	3	0	14	117	12.0%
*Signa Vitae*	0	0	0	0	0	8	0	0	0	0	0	5	0	13	64	20.3%
*Ulusal Travma ve Acil Cerrahi Dergisi*	0	0	0	0	1	0	0	0	1	0	0	0	0	2	150	1.3%
*Unfallchirurg*	0	0	0	0	0	0	1	0	0	0	0	0	2	3	194	1.5%
*Western Journal of Emergency Medicine*	0	0	1	0	15	0	20	0	15	0	15	0	0	66	217	30.4%
*World Journal of Emergency Medicine*	0	0	0	0	0	0	0	0	1	0	0	2	0	3	50	6.0%
*World Journal of Emergency Surgery*	0	0	0	3	2	2	1	0	0	0	0	0	0	8	59	13.6%
Subtotal	0	0	1	9	27	67	96	73	65	80	77	78	192	765	7,195	10.6%

*Jan.*, January; *Feb.*, February; *Mar.*, March; *Apr.*, April; *Jun*., June; *Jul.*, July; *Aug*., August; *Sep*., September; *Oct.*, October; *Nov*., November; *Dec*., December; *BMC*, Boston Medical Center.

**Table 2:** Leading countries, affiliations, and authors of coronavirus disease 2019 publications (publication numbers in brackets).

	Country	Affiliation	Author
1	United States (286)	Harvard Medical School (27)	Elkbuli, A. (17)
2	Italy (50)	University of Toronto (24)	McKenney, M. (17)
3	Canada (46)	Massachusetts General Hospital (19)	Long, B. (12)
4	United Kingdom (45)	Kendall Regional Medical Center (18)	Gottlieb, M. (11)
4	Spain (44)	Monash University (18)	Mitchell, R.D. (10)

**Table 3:** Times-cited analysis of coronavirus disease 2019 publications.

Publication types	Publication numbers	Total cited times	Average cited times	Maximum cited times	Publications cited (%)
Article	373	1,170	3.137	118	175 (46.9)
Letter	229	435	1.900	45	98 (42.8)
Review	67	418	6.239	118	39 (58.2)
Note	59	148	2.508	23	26 (44.1)
Editorial	32	46	1.438	10	14 (43.8)
Short Survey	3	0	0.000	0	0 (0)
Erratum	2	0	0.000	0	0 (0)
Total	765	2,217	2.898		352 (46.0)
