# Supplementary material for: COVID-19 Literature Published in Emergency Medicine Journals in 2020
Source: West J Emerg Med. 2022 May 5;23(3):432–8. doi: 10.5811/westjem.2022.1.55029 (PMC9183767; doi:10.5811/westjem.2022.1.55029)
Supplement: Supplementary file 2 [file wjem-23-432-s002.docx]

# Appendix

**Appendix A.** Journals of the 2019 JCR category of emergency medicine ranked by JCR impact factor. *ISSN: International Standard Serial Number.

| **Rank** | **Journal title** | **ISSN*** |
| --- | --- | --- |
| 1 | Annals of emergency medicine | 0196-0644 |
| 2 | Resuscitation | 0300-9572 |
| 3 | World journal of emergency surgery | 1749-7922 |
| 4 | Emergencias | 1137-6821 |
| 5 | Burns & trauma | 2321-3868 |
| 6 | Academic emergency medicine | 1069-6563 |
| 7 | Emergency medicine journal | 1472-0205 |
| 8 | Scandinavian journal of trauma resuscitation & emergency medicine | 1757-7241 |
| 9 | Prehospital emergency care | 1090-3127 |
| 10 | European journal of emergency medicine | 0969-9546 |
| 11 | European journal of trauma and emergency surgery | 1863-9933 |
| 12 | Injury-international journal of the care of the injured | 0020-1383 |
| 13 | American journal of emergency medicine | 0735-6757 |
| 14 | Western journal of emergency medicine | 1936-900X |
| 15 | World journal of emergency medicine | 1920-8642 |
| 16 | Canadian journal of emergency medicine | 1481-8035 |
| 17 | Emergency medicine Australasia | 1742-6731 |
| 18 | Emergency medicine clinics of north America | 0733-8627 |
| 19 | BMC emergency medicine | 1471-227X |
| 20 | Journal of emergency nursing | 0099-1767 |
| 21 | Prehospital and disaster medicine | 1049-023X |
| 22 | Journal of emergency medicine | 0736-4679 |
| 23 | Pediatric emergency care | 0749-5161 |
| 24 | Emergency medicine international | 2090-2840 |
| 25 | Unfallchirurg | 0177-5537 |
| 26 | Ulusal travma ve acil cerrahi dergisi-turkish journal of trauma & emergency surgery | 1306-696X |
| 27 | Australasian emergency care | 2588-994X |
| 28 | Notfall & rettungsmedizin | 1434-6222 |
| 29 | Notarzt | 0177-2309 |
| 30 | Signa vitae | 1334-5605 |
| 31 | Hong Kong journal of emergency medicine | 1024-9079 |

**Appendix B.** Search terms for COVID-19 publications and total emergency medicine journal publications in 2020.

**Appendix B.1.** The search terms for COVID-19 publications in 2020.

ISSN(0196-0644) OR ISSN(0300-9572) OR ISSN(1749-7922) OR ISSN(1137-6821) OR ISSN(2321-3868) OR ISSN(1069-6563) OR ISSN(1472-0205) OR ISSN(1757-7241) OR ISSN(1090-3127) OR ISSN(0969-9546) OR ISSN(1863-9933) OR ISSN(0020-1383) OR ISSN(0735-6757) OR ISSN(1936-900X) OR ISSN(1920-8642) OR ISSN(1481-8035) OR ISSN(1742-6731) OR ISSN (0733-8627) OR ISSN(1471-227X) OR ISSN(0099-1767) OR ISSN(1049-023X) OR ISSN(0736-4679) OR ISSN(0749-5161) OR ISSN(2090-2840) OR ISSN(0177-5537) OR ISSN(1306-696X) OR ISSN(2588-994X) OR ISSN(1434-6222) OR ISSN(0177-2309) OR ISSN(1334-5605) OR ISSN(1024-9079) AND PUBYEAR > 2019 AND PUBYEAR < 2021 AND TITLE-ABS-KEY(COVID)

**Appendix B.2.** The search terms for SARS but not COVID-19 publications in 2020.

ISSN ( 0196-0644 ) OR ISSN ( 0300-9572 ) OR ISSN ( 1749-7922 ) OR ISSN ( 1137-6821 ) OR ISSN ( 2321-3868 ) OR ISSN ( 1069-6563 ) OR ISSN ( 1472-0205 ) OR ISSN ( 1757-7241 ) OR ISSN ( 1090-3127 ) OR ISSN ( 0969-9546 ) OR ISSN ( 1863-9933 ) OR ISSN ( 0020-1383 ) OR ISSN ( 0735-6757 ) OR ISSN ( 1936-900x ) OR ISSN ( 1920-8642 ) OR ISSN ( 1481-8035 ) OR ISSN ( 1742-6731 ) OR ISSN ( 0733-8627 ) OR ISSN ( 1471-227x ) OR ISSN ( 0099-1767 ) OR ISSN ( 1049-023x ) OR ISSN ( 0736-4679 ) OR ISSN ( 0749-5161 ) OR ISSN ( 2090-2840 ) OR ISSN ( 0177-5537 ) OR ISSN ( 1306-696x ) OR ISSN ( 2588-994x ) OR ISSN ( 1434-6222 ) OR ISSN ( 0177-2309 ) OR ISSN ( 1334-5605 ) OR ISSN ( 1024-9079 ) AND PUBYEAR > 2019 AND PUBYEAR < 2021 AND TITLE-ABS-KEY ( SARS ) AND NOT TITLE-ABS-KEY(COVID)

**Appendix B.3.** The search terms for total emergency medicine journal publications in 2020.

ISSN(0196-0644) OR ISSN(0300-9572) OR ISSN(1749-7922) OR ISSN(1137-6821) OR ISSN(2321-3868) OR ISSN(1069-6563) OR ISSN(1472-0205) OR ISSN(1757-7241) OR ISSN(1090-3127) OR ISSN(0969-9546) OR ISSN(1863-9933) OR ISSN(0020-1383) OR ISSN(0735-6757) OR ISSN(1936-900X) OR ISSN(1920-8642) OR ISSN(1481-8035) OR ISSN(1742-6731) OR ISSN (0733-8627) OR ISSN(1471-227X) OR ISSN(0099-1767) OR ISSN(1049-023X) OR ISSN(0736-4679) OR ISSN(0749-5161) OR ISSN(2090-2840) OR ISSN(0177-5537) OR ISSN(1306-696X) OR ISSN(2588-994X) OR ISSN(1434-6222) OR ISSN(0177-2309) OR ISSN(1334-5605) OR ISSN(1024-9079) AND PUBYEAR > 2019 AND PUBYEAR < 2021
